# Governance of the wildlife trade and the prevention of emerging zoonoses: a mixed methods network analysis of transnational organisations, silos, and power dynamics

**DOI:** 10.1186/s12992-024-01055-7

**Published:** 2024-06-20

**Authors:** Chloe Clifford Astbury, Anastassia Demeshko, Eduardo Gallo-Cajiao, Ryan McLeod, Mary Wiktorowicz, Cécile Aenishaenslin, Katherine Cullerton, Kirsten M. Lee, Arne Ruckert, A. M. Viens, Peter Tsasis, Tarra L. Penney

**Affiliations:** 1 Global Food Systems & Policy Research, School of Global Health, York University, Toronto, ON Canada; 2 Dahdaleh Institute for Global Health Research, York University, Toronto, ON Canada; 3 Global Strategy Lab, York University, Toronto, ON Canada; 4 School of Marine and Environmental Affairs, University of Washington, Seattle, WA USA; 5 Department of Human Dimensions of Natural Resources, Warner College of Natural Resources, Colorado State University, Fort Collins, CO USA; 6 School of Health Policy and Management, York University, Toronto, ON Canada; 7 Faculté de médecine vétérinaire, Université de Montréal, Montréal, Québec Canada; 8 Centre de recherche en santé publique de l’Université de Montréal et du CIUSSS du Centre-Sud-de-l’Île- de-Montréal, Montréal, Québec Canada; 9 School of Public Health, University of Queensland, Brisbane, QLD Australia; 10 School of Epidemiology and Public Health, University of Ottawa, Ottawa, Canada

**Keywords:** Emerging zoonoses, Wildlife trade, Global governance, One Health, Transnational organisations, Social network analysis

## Abstract

**Introduction:**

The wildlife trade is an important arena for intervention in the prevention of emerging zoonoses, and leading organisations have advocated for more collaborative, multi-sectoral approaches to governance in this area. The aim of this study is to characterise the structure and function of the network of transnational organisations that interact around the governance of wildlife trade for the prevention of emerging zoonoses, and to assess these network characteristics in terms of how they might support or undermine progress on these issues.

**Methods:**

This study used a mixed methods social network analysis of transnational organisations. Data were collected between May 2021 and September 2022. Participants were representatives of transnational organisations involved in the governance of wildlife trade and the prevention of emerging zoonoses. An initial seed sample of participants was purposively recruited through professional networks, and snowball sampling was used to identify additional participants. Quantitative data were collected through an online network survey. Measures of centrality (degree, closeness, and betweenness) were calculated and the network’s largest clique was identified and characterised. To understand the extent to which organisations were connected across sectors, homophily by sector was assessed using exponential random graph modelling. Qualitative data were collected through semi-structured interviews. The findings from the quantitative analysis informed the focus of the qualitative analysis. Qualitative data were explored using thematic analysis.

**Results:**

Thirty-seven participants completed the network survey and 17 key informants participated in semi-structured interviews. A total of 69 organisations were identified as belonging to this network. Organisations spanned the animal, human, and environmental health sectors, among others including trade, food and agriculture, and crime. Organisation types included inter-governmental organisations, non-governmental organisations, treaty secretariats, research institutions, and network organisations. Participants emphasised the highly inter-sectoral nature of this topic and the importance of inter-sectoral work, and connections were present across existing sectors. However, there were many barriers to effective interaction, particularly conflicting goals and agendas. Power dynamics also shaped relationships between actors, with the human health sector seen as better resourced and more influential, despite having historically lower engagement than the environmental and animal health sectors around the wildlife trade and its role in emerging zoonoses.

**Conclusion:**

The network of transnational organisations focused on the governance of wildlife trade and the prevention of emerging zoonoses is highly multi-sectoral, but despite progress catalysed by the COVID-19 pandemic, barriers still exist for inter-sectoral interaction and coordination. A One Health approach to governance at this level, which has gained traction throughout the COVID-19 pandemic, was shared as a promising mechanism to support a balancing of roles and agendas in this space. However, this must involve agreement around equity, priorities, and clear goal setting to support effective action.

**Supplementary Information:**

The online version contains supplementary material available at 10.1186/s12992-024-01055-7.

## Introduction

Recent and ongoing global health crises, including the COVID-19 pandemic and outbreaks of mpox and Ebola, have highlighted the urgent threat presented by emerging zoonoses [1]. Many policy responses to emerging zoonoses have focused on controlling human-to-human transmission, but leading organisations and experts, including the United Nations Environment Programme, the International Livestock Research Institute, and the Intergovernmental Science-Policy Platform on Biodiversity and Ecosystem Services, have called for a greater focus on the drivers of zoonotic disease emergence in both animal and human populations [1–6]. This approach, sometimes called deep prevention, would need to target upstream drivers to reduce the risk of outbreaks occuring [7]. However, consensus on strategies to foster prevention of emerging zoonoses among transnational organisations are in the early stages of development.

Within this approach, the wildlife trade is a key arena for intervention [8]. In this analysis, we define wildlife trade broadly, as including domestic or international and legal or illegal trade of specimens originally sourced from the wild, traded alive or dead, involving whole individuals or body parts across all life stages for various uses including food, fashion, medicine, zoos and pets [9]. The wildlife trade may contribute to the risk posed by emerging zoonoses through numerous pathways, including human exposure to zoonotic pathogens through wildlife consumption and handling; transmission of pathogens from rural areas to densely populated urban ones in order to sell wildlife specimens; transmission of pathogens within and between countries and regions when animals are sold and transported through trade networks; increased contact between animal species (including both wild and domesticated animals) across various stages of wildlife trade; and changes in wildlife population dynamics with knock-on effects for reservoir populations, habitats, and disease ecology [8, 10, 11].

Despite the importance of interventions targeting the wildlife trade for reducing risk from emerging zoonoses, effective and coordinated action in this area is challenging. First, responsibility for the governance of wildlife trade spans multiple sectors, including food security and safety, economic development and biodiversity conservation, and the issue has additional relevance for sectors including trade, as well as human and animal health [12]. The goals of these sectors may sometimes be in conflict, meaning that approaches to governing the wildlife trade must be carefully negotiated to agree on reasonable trade-offs. Second, the global dimension of the wildlife trade – with some wildlife and wildlife products being traded across national borders – makes interaction between countries essential to effective intervention. Finally, international collaboration can help strengthen domestic capacity for wildlife trade governance through policy development, implementation support and sharing expertise and best practice. The inter-sectoral and international nature of this issue makes interaction crucial, and transnational organisations may play an important role in supporting this.

In addressing cross-sectoral problems, such as the wildlife trade, a One Health perspective, which recognises the health of humans, animals, and the environment as closely linked and inter-dependent [13], can strengthen policy and governance approaches. Building on this perspective, a call has been made by experts and organisations including the World Health Organisation, the Food and Agriculture Organisation, the World Organisation for Animal Health, and the United Nations Environment Programme for a more coordinated and collaborative approach at the international level to prevent the spillover of zoonotic pathogens in human populations [14, 15].

While this call for more coordinated and collaborative action has been issued by intergovernmental organisations that set international guidelines and standards and support member states through training and other measures, the nature of the existing network of organisations working on the governance of the wildlife trade at the transnational level has not been characterised. The extent of interaction between organisations and sectors to reduce the risk of zoonotic disease emergence from the wildlife trade is unknown. Although the value of interaction is acknowledged, many parts of the global health landscape are fragmented for reasons including the large and growing number of actors; lack of centralised leadership; and competing interests [16]. This problem may be even more severe in cross-sectoral contexts, such as wildlife trade governance and the prevention of emerging zoonoses [12].

### Aims and scope

The aim of this study is to characterise the structure and function of the global network of transnational organisations that interact as part of the governance of the wildlife trade, and to assess these network characteristics in terms of how they might support or undermine effective progress on the prevention of emerging zoonoses.

## Methods

### Approach and design

This analysis consisted of a mixed methods social network analysis (MMSNA) of the network of transnational organisations contributing to the governance of wildlife trade (including legal and illegal trade conducted for all uses). Networks are simultaneously structure (i.e., a combination of connections between elements), process (i.e., a way in which these elements and connections evolve in response to each other), and function (i.e., the outcomes these elements and connections are oriented towards the extent to which they achieve them), and can therefore be usefully characterised using both quantitative and qualitative approaches [17]. Qualitative data can provide information on how the network is evolving, as well as rich contextual information about the relationships and internal workings of structures identified through quantitative approaches.

**Fig. 1:**
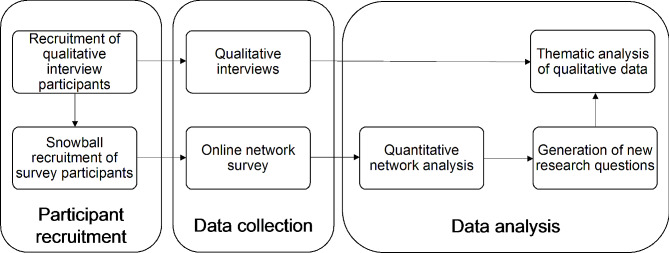
Methodology flow chart illustrating participant recruitment, data collection and data analysis

We employed an MMSNA to understand the structure, processes, and function of this network. MMSNA takes a number of forms, with applications differing in how they integrate quantitative and qualitative data [17–19]. In particular, the purpose of integrating these two types of datasets may vary, informing how and when data is integrated. In this study, we implemented an explanatory sequential design [20], using quantitative network analysis to inform the analysis of qualitative data (Fig. 1). Through this approach, we aimed to develop a deeper understanding of the functions and processes underlying our structural findings [21].

Participant recruitment and data collection started in May 2021 and ended in September 2022.

The study was approved by the Human Participants Review Sub-Committee of York University’s Ethics Review Board (certificate #e2020-310) and conforms to the standards of the Canadian Tri-Council Research Ethics guidelines. Interview participants consented to participate either verbally or in writing before the start of the interview. Survey participants indicated their consent using an online form before starting the survey.

### Participant recruitment

This study took a relational approach to drawing the boundaries of the network [22], starting from a seed sample and asking participants to identify additional actors who they thought belonged to the network. All participants were identified via professional networks and desktop searches for relevant organisations, as well as snowball sampling, and recruited by email.

All participants were invited to complete the survey, but only a subset of participants were invited to participate in interviews.

Interview participants were key informants based in transnational organisations (i.e., organisations that operate in a way that transcends national borders [23]), primarily in intergovernmental organisations (i.e., UN agencies or other bodies established through agreements between national governments) and treaty secretariats, involved in issues relating to the governance of the wildlife trade, human health, animal health, international trade and food security, and the prevention of emerging zoonoses. We selected interview participants based on the relevance of their expertise and roles to our research aims, and we stopped interview recruitment and data collection when we reached data saturation [24]. Interview participants served as a seed sample: each participant was asked to share organisation names and contact details of professionals that they interacted with around the governance of the wildlife trade and the prevention of emerging zoonoses at other organisations. Potential participants were emailed up to three times with an invitation to participate. Where organisation names were provided without contact details, additional contacts based at named organisations were identified through desktop searches and invited to participate via email or social media.

Organisations were considered to be the actors in this network (i.e., the unit of analysis), and recruitment strategies for the survey targeted one representative from each organisation, rather than attempting to recruit all potential individuals working in this area. Depending on the size of the organisation and the breadth of its scope, we recruited participants in a leadership position for the organisation as a whole, or for the portion of its work that was most closely linked to the governance of the wildlife trade. We stopped survey recruitment and data collection when we reached data saturation (i.e., survey responses ceased to identify additional organisations), and at least one contact at each identified organisation had been invited to participate three times. In introducing the survey to participants, we emphasised that we were interested in the interactions of the organisation as a whole, and invited potential participants to forward the survey to a colleague who might be best placed to speak from the organisational perspective, if they did not feel able to do so, and advised participants that they could gather input from colleagues to complete the survey if they felt this was appropriate.

In a small number of cases, more than one participant was recruited from a single organisation, where participants referred a colleague within their own organisation as an additional participant to provide a fuller picture of their organisation’s activities. In this case, data from those participants were combined as representing the range of interactions and activities undertaken by an organisation.

### Data collection

The data set consisted of survey and semi-structured interview data.

Survey data was collected through the online survey platform SmartSurvey [25]. Participants were asked to identify their own organisation as well as other organisations they interacted with around wildlife trade governance and the prevention of emerging zoonoses. Specifically, they responded to the questions: “Do you interact with anyone at any of the following organisations around the governance of wildlife trade and/or the prevention of emerging zoonoses?” (participants could select as many organisations as they wanted from a list) and “Do you interact with anyone at any organisations other than those listed above around the governance of wildlife trade and/or the prevention of emerging zoonoses?”.

Interview data was collected through semi-structured key informant interviews by MW and EGC. Each interview lasted approximately an hour and was conducted through the online video platform Zoom [26]. Interview questions focused on topics including international wildlife trade management; response to zoonotic disease outbreaks and pandemics; and coordination in efforts to govern the wildlife trade and reduce related public health risks. Interviews were audio recorded and transcribed verbatim.

Where participants completed both the survey and the interview, the survey was completed after the interview, with participants being sent a link to the survey to complete it in their own time.

Based on a review of organisational websites, organisations were classified by two characteristics to facilitate analysis. First, organisations were classified by the sector to which they belonged (Table 1), including organisations working in each of the three sectors typically associated with the concept of One Health (animal health, human health, environmental health) [13, 27], organisations working in other sectors, and ‘One Health’ organisations. Organisations were also classified by organisation type adapted from an existing typology of organisational actors in global health: consultancy; government department; inter-governmental organisation; network; non-governmental organisation; professional association; regional economic initiative; research institution; trade association; treaty secretariat; voluntary partnership secretariat [28].

**Table 1:** Definition of organisational sectors

Sector	Definition
Human health	Public and population health; healthcare; human medicine
Animal health	Veterinary public health and medicine for livestock, companion animals and/or wildlife
Environmental health	Environmental or ecosystem protection or conservation
Other	Organisations not specific to human, animal, or environmental health (e.g., trade, food, agriculture, crime, border control, travel, development)
One Health	While definitions of One Health typically include three dimensions (animal, human, and environmental health) [27], for the purposes of this analysis, organisations were classified as One Health if they self-identified as a One Health organisation or if their work spanned two or more of the above sectors

### Analysis

First, we analysed quantitative data by calculating network statistics developed in the field of social network analysis [29]. We considered the network to be a binary, undirected network [29]. We analysed survey data in R [30]. We used the igraph package [31] to calculate nodal properties and identify sub-groups, the statnet [32] and ergm [33] packages to assess homophily and the ggraph package [34] to visualise the network.

We evaluated the *centrality* of nodes – in our case, organisations – within the network, which was treated as undirected. Centrality assesses the extent to which organisations are involved in network relationships, and can be characterised in different ways [35]. We calculated three measures of centrality within this network: [35]

- **Degree centrality**: How many organisations a given organisation is directly connected to, signifying how active and well-connected that organisation is within the network;
- **Betweenness centrality**: How often an organisation appears within the shortest path *between* organisations, indicating the extent to which that organisation can act as a gatekeeper or broker in terms of the flow of information or resources within the network; and.
- **Closeness centrality**: How close (i.e., how many relationship steps away) an organisation is to other organisations, indicating independence or efficiency as an actor within the network, as the organisation does not depend on others to connect or communicate with partners [29].

As well as assessing the centrality of particular nodes within the network, we also identified *cliques*: sub-groups of three or more organisations within the network, all members of which are connected to one another [29, 35]. Members of one or more cliques tend to be core members within the network. We therefore identified the largest clique, as well as the organisations belonging to the greatest number of cliques.

We also assessed *homophily* within the network: the tendency of nodes to be connected to nodes with similar traits. We assessed whether organisations from the same sector (animal health, human health, environment, or One Health) were more likely to be connected to organisations from the same sector, in order to assess the extent to which the network is characterised by cross-sectoral interaction. To assess network homophily, we used exponential random graph modelling (ERGM), a modelling approach which takes into account key characteristics of network data, particularly that the observations within a network are highly inter-dependent, and that network sampling is purposive rather than probability-based [29]. ERGM has been explained in greater detail elsewhere [36]. Briefly, ERGM relies on the generation of networks with the same structural properties as the observed network (known as ‘degree randomisation’), creating a distribution of possible networks. The observed network is then compared to this distribution, allowing a range of hypotheses about the network’s properties to be tested. In this case, we used ERGM to assess whether the observed homophily by sector was higher than what was likely to be explained by network structure alone, after adjusting for organisation type.

Second, we analysed qualitative interview data. Based on our MMSNA approach, using qualitative data to investigate ideas suggested by quantitative findings, we developed research questions to explore in the qualitative data. Thematic analysis was conducted using the approach described by Braun and Clarke where the focus is guided by the researcher’s analytic interests [37], with the four research questions informed by the quantitative findings serving as an a priori coding framework. We subsequently identified sub-themes through close reading and inductive coding of the transcripts. Thematic analysis was conducted by three researchers working together (CCA, AD and RM), using an iterative process to develop meaning through discussion and repeated review of the data and codes [38]. To support this process, we used the collaborative qualitative data analysis software Dedoose [39].

## Results

### Sample characteristics

Semi-structured interviews were conducted with an initial sample of 17 key informants from 14 organisations identified through professional networks. By pursuing the network connections of these interview participants, 69 organisations were identified as part of the network of transnational organisations focused on the governance of wildlife trade and the prevention of emerging zoonoses. Of the 133 potential participants invited (covering all 69 organisations), 37 participants from 33 organisations completed the network survey (with respondents from two larger organisations suggesting multiple participants to represent the breadth of their work). At the organisation level, this resulted in a response rate of 48% (33/69 organisations in the network). Response rates were slightly higher for organisations in the animal health and One Health sectors (45% in human health, 60% in animal health, 43% in environmental health, 63% in One Health, 53% in other). For organisation types, response rates from networks were highest, while response rates from non-governmental organisations were lowest (58% in intergovernmental organisations, 39% in non-governmental organisation, 60% in agreement secretariats, 71% in networks, 45% in research institutions, and 55% in other, including organisations such as consultancies, government departments and professional organisations). Characteristics of organisations identified within the network are described in Table 2. Characteristics of interview participants are described in Table 3.

**Table 2:** Characteristics of organisations in the network (*n* = 69)

Type	Sector					
	Animal health	Human health	Environmental health	One Health	Other	Total (*n* (%))
Consultancy	0	0	1	0	0	1 (1)
Government department	0	0	1	0	0	1 (1)
Inter-governmental organisation	1	2	2	0	7	12 (17)
Network	1	1	0	5	0	7 (10)
Non-governmental organisation	5	14	2	1	1	23 (33)
Professional association	3	0	3	0	1	7 (10)
Regional economic initiative	0	0	0	0	1	1 (1)
Research institution	2	2	1	1	5	11 (16)
Trade association	0	0	0	0	1	1 (1)
Treaty secretariat	0	3	0	0	0	3 (4)
Voluntary partnership secretariat	0	1	0	1	0	2 (3)
Total (n (%))	12 (17)	23 (33)	10 (15)	8 (12)	16 (23)	69 (100)

**Table 3:** Characteristics of interview participants (*n* = 17)

Type	Sector					
	Animal health	Human health	Environmental health	One Health	Other	Total (*n* (%))
Inter-governmental organisation	1	2	1	0	4	8 (47)
Network	0	0	0	1	0	1 (6)
Non-governmental organisation	1	0	2	0	0	3 (18)
Professional association	1	0	0	0	0	1 (6)
Research institution	1	0	0	0	0	1 (6)
Treaty secretariat	0	0	3	0	0	3 (18)
Total (n (%))	4 (24)	2 (12)	6 (35)	1 (6)	4 (24)	17 (100)

### Network description and organisation centrality

**Fig. 2:**
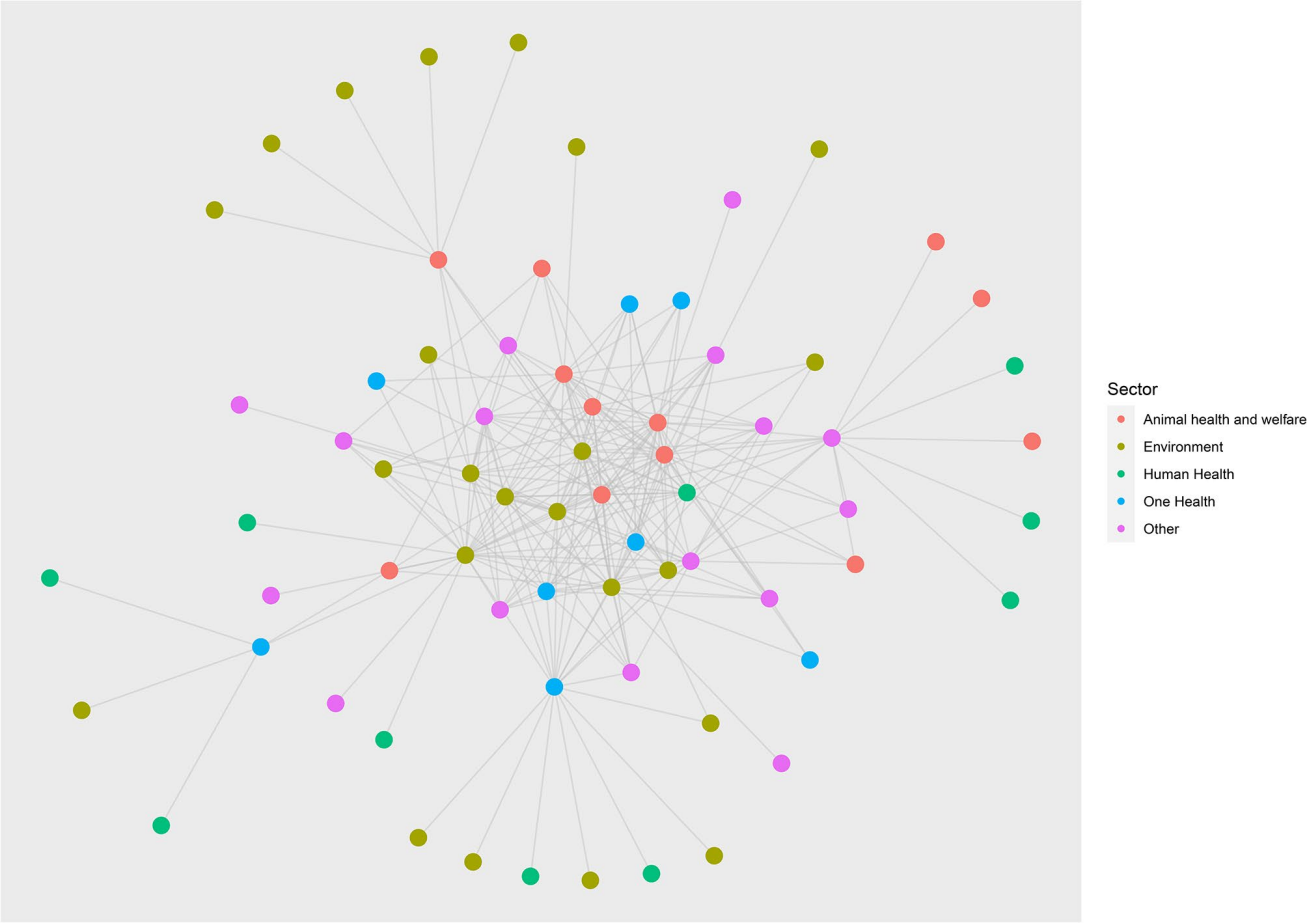
Network mapping of organisations involved in the prevention of emerging zoonoses and the management of the wildlife trade. Node colour indicates sector. The figure highlights the lack of centrality of organisations in the human health sector, despite their relatively high number

**Fig. 3:**
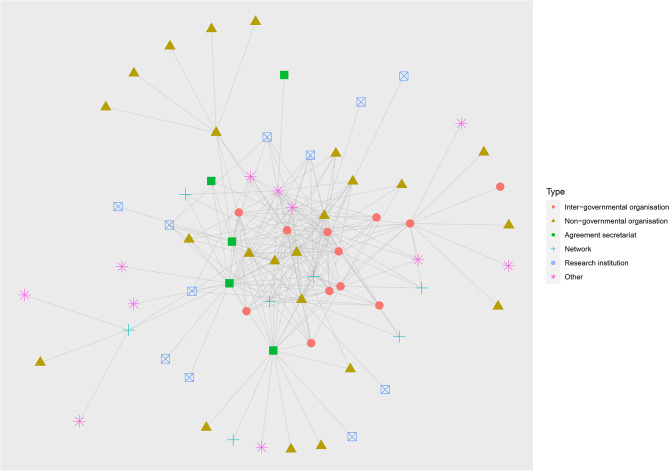
Network mapping of organisations involved in the prevention of emerging zoonoses and the management of the wildlife trade. Node colour and shape indicates organisation type. Organisation types have been grouped together for easier visualisation (treaty secretariat, voluntary agreement secretariat = agreement secretariat; consultancy, government department, professional association, regional economic initiative, trade association = other.) Inter-governmental organisations have relatively high centrality

The identified network was composed of 280 connections (characterised here as interactions identified by survey participants) between 69 organisations (Fig. 2 by organisation sector and Fig. 3 by organisation type). The network’s diameter (the length of the longest path between two organisations) is 5. The average degree (average number of connections per organisation) is 4.06.

Organisations varied in terms of degree, betweenness and closeness centralities (organisations with the highest degree, betweenness and closeness centralities in Table 4; all centrality information in Supplementary File 1). While a substantial number of organisations in the network focused on human health (33%, see Table 2), organisations with high centrality were predominantly focused on One Health, animal health, and environmental health. This suggests that, while many organisations focused on human health are included in the network, organisations in other sectors, particularly animal health, environmental health and One Health, are more connected with multiple organisations across it, having higher degree, betweenness and closeness centrality. Inter-governmental organisations showed high centrality across all three measures, suggesting that these organisations are key actors within the network. Two of the seven network organisations are situated within the top ten organisations for some measures of centrality. This may suggest that some of these network associations have been more successful than others in connecting previously unconnected organisations.

**Table 4:** Organisations with the highest degree, betweenness and closeness centralities

Rank	Sector	Type	ID	Degree
1	Animal health	Inter-governmental organisation	1	30
2	Environmental health	Inter-governmental organisation	2	28
3	Other	Inter-governmental organisation	3	23
4	Environmental health	Non-governmental organisation	4	22
5	Animal health	Network	5	21
6	Environmental health	Non-governmental organisation	6	21
7	One Health	Research institution	7	20
8	Other	Inter-governmental organisation	8	20
9	Environmental health	Treaty secretariat	9	19
10	Environmental health	Inter-governmental organisation	10	19
**Rank**	**Sector**	**Type**	**ID**	**Betweenness**
1	Environmental health	Inter-governmental organisation	2	561
2	One Health	Network	11	412
3	Animal health	Professional association	15	394
4	Other	Non-governmental organisation	21	325
5	Animal health	Inter-governmental organisation	1	241
6	Other	Inter-governmental organisation	8	216
7	One Health	Research institution	7	215
8	One Health	Voluntary partnership secretariat	32	198
9	Environmental health	Treaty secretariat	9	193
10	Other	Inter-governmental organisation	2	159
**Rank**	**Sector**	**Type**	**ID**	**Closeness**
1	One Health	Research institution	7	0.0084
2	Animal health	Inter-governmental organisation	2	0.0083
3	Environmental health	Inter-governmental organisation	1	0.0083
4	Other	Inter-governmental organisation	8	0.0083
5	Other	Inter-governmental organisation	9	0.0078
6	Environmental health	Non-governmental organisation	3	0.0078
7	Environmental health	Treaty secretariat	4	0.0078
8	Animal health	Network	5	0.0078
9	Human Health	Inter-governmental organisation	12	0.0077
10	Environmental health	Inter-governmental organisation	10	0.0076

### Clique identification

The largest clique in the network was composed of ten organisations. This was dominated by inter-governmental organisations (*n* = 6), but also included two non-governmental organisations, one research institution, and one network organisation. A range of sectors were represented. (One Health *n* = 1, environmental health *n* = 4, animal health *n* = 2, development *n* = 1, food and agriculture *n* = 1, human health *n* = 1), although environmental health dominated. The organisations in this clique also appeared as frequent members of other, smaller cliques. This suggests that a core group of organisations, particularly inter-governmental organisations, work on these topics together, which may allow them to share information, resolve issues and reach consensus on how to move forward within a sub-group, which could be shared across the larger network to facilitate collective approaches and action.

### ERGM to assess homophily by sector

Table 5 shows the results of the ERGM testing homophily by sector. Model results do not support the hypothesis that organisations from the same sector are more likely to be connected.

**Table 5:** Cross-sectional exponential random graph model predicting organisational interaction based on homophily for sector, adjusted for organisation type

	Estimate (SE)
**Endogenous organising factors**	
Edges	-1.86 (0.26)*
**Homophily effects**	
Animal health	0.64 (0.39)
Environmental health	-0.09 (0.30)
Human health	0.29 (1.09)
One Health	-0.44 (0.68)
Other	0.06 (0.40)

*P < 0.0001

### Research questions informed by quantitative findings

The network mapping and statistics informed the development of four additional research questions about the functioning of the network. These were explored through thematic analysis of semi-structured interview data:

- Are there sectoral differences in how active, independent, and powerful organisations are within this network?
- How have network organisations impacted the network’s ability to interact effectively?
- How does the core group of organisations identified (i.e., the largest clique identified through the quantitative social network analysis) impact interaction across the network?
- Are there differences in how organisations interact within versus outside their own sector?

### Are there sectoral differences in how active, independent, and powerful organisations are within this network?

While participants identified a will to collaborate across sectors, they also highlighted some competition for resources and tension in terms of different goals:

*“I mean, so in other words, whilst we [sectoral organisations] have separate goals, […] if we’re centralised we’ll always have a different perspective, there will always be conflict. And whoever is stronger and more dominant, will essentially win. […] Until you get around the table in a balanced way, it will always shift to the strongest sector.”*

(Key informant, animal health)

Overall, the power and resources held by the human health sector were repeatedly emphasised, despite its relatively low centrality within this network. This was discussed at the international level with reference to governance structures such as the Tripartite (now the Quadripartite) [13] and the ability to put in place legal instruments such as the International Health Regulations (IHR): [40]

*“I mean you know the Tripartite is something, it’s good, but the honest truth in my opinion about the Tripartite is it’s been doing two-thirds health.”*

(Key informant, other)

“Yes, IHR is interesting. And of course, it comes back to the power issue. So they’ve been able to introduce a regulatory framework that is very helpful if you’re dealing with human infection, [but] it’s almost impossible to get a diagnosis very often in wildlife, because the regulatory framework, for example, moving samples around the world are so complicated, so difficult.”

(Key informant, animal health)

The animal health and, in particular, the environmental health sectors, were seen as less well-resourced, and tended to be less dominant in decision-making processes:

*“The environment sector is under-resourced and can’t take on yet another challenge.”* (Key informant, One Health).

However, the specific topics on which this network focused may explain the centrality of the animal and environmental health sectors. Organisations focused on human health did not always see the relevance of the wildlife trade, the prevention of emerging zoonoses or related issues such as environmental protection to their organisational remit:

“We talk to the health ministry well and they find it interesting what we do, but somehow they still think that we’re keeping animals healthy and it has nothing to do with them.”

(Key informant, One Health)

In some cases, this made the human health sector less likely to engage on these topics. While it was perceived that some actors in the human and animal health sectors acknowledged the importance of the environment to health, this did not necessarily translate to a sense of responsibility for environmental issues:

“And sometimes it’s easier to convince people in the health sector that the [environment] plays an important role for health, and changes within the environment do influence the health of people and animals. So that’s sometimes easier to get across to get the health sector convinced, but then they don’t feel responsible, you know? So if it’s major conservation we don’t do that. Or even climate mitigation or adaptation, that’s not for us.”

(Key informant, One Health)

However, many key informants emphasised the role that paradigms such as One Health and planetary health, as well as health crises such as the COVID-19 pandemic, had played in highlighting the relevance of these topics for human health, and building more interest and enthusiasm from the human health sector:

“And increasingly that’s very much going into strengthening the interaction with partner agencies is moving into the prevention part of it because, for example, the current pandemic has shown us the limitation of how prepared can we be and how we can respond to these type of events showing that it was clear this time that once these new pathogens are out of the box so to speak it’s simply too late to control them. So being able to do more the prevention is something we want to investigate in the future.”

(Key informant, human health)

Key informants also emphasised that in some cases the human health sector *needed* the animal health sector to work in the area of emerging zoonoses, as animal health experts typically had more extensive knowledge of pathogens that were common in animals before being transmitted more frequently to humans. These instances could foster equitable relationships and interaction:

*“And often veterinary services are seen as a lesser partner sometimes by public health [but avian] influenza was a good example, from my experience, because the public health sector really needed us. They needed the viruses, they needed the information from the animal health sector to inform public health strategy. And very importantly, to develop a vaccine if they needed to. So it was a very equitable relationship.”*

(Key informant, animal health)

Conversely, the opportunities presented by the human health sector’s platform and resources were also emphasised by some participants: the involvement of this sector had the potential to raise the profile of issues relating to the wildlife trade and the governance of emerging zoonoses.

While there were sectoral differences in power and investment across the network, many participants saw the One Health approach, which was seen to have increasingly gained traction during the COVID-19 pandemic, as a mechanism to give the animal and environmental health sectors more of a seat at the table. However, many cautioned that this approach could become tokenistic unless it was based on specific goals shared by the sectors, and supported by a clear plan for implementation.

### How have network organisations impacted the network’s ability to interact effectively?

Key informants’ perspectives aligned with the quantitative finding that network organisations (i.e., organisations whose explicit goal is to bring actors together) were not playing a key role in creating new connections between organisations in the network. Participants reported that they had had some interactions with many of the organisations in the network for many years, independent of the network organisations, some of which had only emerged fairly recently.

However, network organisations were seen to support interaction in other ways. Network organisations contributed to centring previously marginalised interests, such as the health of wildlife populations, and working to convene parties and facilitate new work and interactions on these topics:

“So this is where we see our role is really as a convenor in some ways. It’s to unite around shared goals with a shared vision. That was something from our experience that was quite important, to develop a joint vision that we can all get behind. And of course that’s a challenge in itself because if it gets too vague then anybody will just kind of align behind it and that was not really unifying either. And so it’s not easy.”

(Key informant, One Health)

The services they provided to their members included highlighting potential areas of synergy, where different organisations could work together on projects that would benefit them both. For example, where multiple organisations were seeking funds for projects with overlap in terms of geography or disease focus, network organisations connected organisations to develop more competitive and resource-efficient funding applications. Network organisations also provided connections to relevant experts and supported partnership development to access and use resources more effectively. They also worked with organisations and governments at the national level, connecting countries to support learning and information sharing. For example, where a national government expressed interest in improving their capacity to conduct risk assessments in wildlife markets, a network organisation organised an information session on the topic, inviting representatives from other countries to attend and share information, resources and lessons from their own efforts in the area.

### How does the core group of organisations impact interaction across the network?

The core group of organisations seemed central to the functioning of the network. They played a convening function, bringing together different organisations both within and between countries through mechanisms such as working groups or expert panels, sharing information, and providing a platform to build buy-in from national-level actors. They also advocated across sectors, making the case for disease in wildlife populations as a shared threat, and highlighting cases where interventions were a ‘win-win’ for multiple sectors. This enabled them to involve new stakeholders and build buy-in. Finally, this core group of organisations had a certain institutional memory of collaborative work, and could draw on existing networks and modes of working that had been put in place during previous outbreaks of zoonotic disease.

However, this core group had certain limitations in its activities. Transnational organisations typically interfaced with their sectoral equivalent within a national context (e.g., a transnational animal health organisation would mainly interact with a national-level animal health organisation). This meant that, while inter-sectoral coordination was happening transnationally, in-country work depended on existing coordination between sectors in a given country context. This inter-sectoral interaction was not always present at the national level:

"[…] over the past year, we’ve been surveying our member countries on to what extent they are involved in regulating wildlife trade. And it varies from country to country. […] And we can have an effect working through veterinary services but only if the veterinary services are a working partnership with other actors at national level, whether that be wildlife, health, or foreign authorities or environment agencies, or whatever."

(Key informant, animal health)

There was also a perception that this core group – dominated by inter-governmental organisations – faced bureaucratic barriers to action and interaction, and may struggle to respond to rapidly changing issues such as the prevention of emerging zoonoses:

“The bureaucratic barrier can hinder efficient collaboration among global government offices due to entrenched working habits, which may take years to change.”

(Key informant, animal health)

Bureaucratic barriers identified by participants included a misalignment between sectoral mandates and responsibilities at national and transnational levels, particularly for an area such as the wildlife trade which may be governed by different sectors in different contexts:

“Or they may not have the mandate – in one country it might be the public health institute that has the mandate for food safety regulations in a food market, in another place it might be an agricultural department, right. So, because wildlife suffers the fate of being of interest to everybody, nobody wants to own it. And because there’s no minister of coordination, right, there is no department of integration, there’s nobody who develops the strategic advantages by working in cross-sectors.”

(Key informant, animal health)

Participants also reported bureaucratic barriers to coordinated transnational action on animal health which were not present in the human health sector. For example, one participant reported challenges in identifying disease outbreaks in animal, and particularly wildlife, populations, due to regulations preventing sending samples across national borders for timely testing. While the International Health Regulations supported these procedures for human populations, the same measures were not in place for wildlife.

In addition, while the core group was cross-sectoral, there was a perception that the learnings or commitments that emerged from their interactions did not necessarily diffuse into their respective organisations. Finally, there was concern that this core group kept certain highly relevant voices out of mainstream decision-making, particularly actors from the environmental health sector: animal and, particularly, human health agencies tended to be more powerful and better funded, and the relevance of the environmental health sector to decision-making was not always recognised, either by the environmental health sector itself or agencies from other sectors.

### Are there differences in how organisations interact within versus outside their own sector?

Key informants reported that the wildlife trade was outside the traditional remit of the animal health sector, which typically focuses on providing veterinary care for domesticated animals, as well as outside the human health sector, despite the risk presented to human health by emerging zoonoses and the relevance of the wildlife trade to human health topics such as food safety. However, many stated that this had changed somewhat because of the COVID-19 pandemic. As a result, the governance of the wildlife trade necessitated inter-sectoral interaction, which may explain the lack of homophily by sector within this network: some level of inter-sectoral interaction was required to address a topic that does not fall clearly into the remit of any one sector.

At the same time, participants highlighted distinctions between intra- and intersectoral interaction. Interaction *across* sectors typically focused on ad hoc connections, such as establishing working groups or developing training for on-the-ground staff that needed a more holistic skill set. This type of work depended on mutual willingness and the recognition of shared aims, as well as the availability of resources to support sustained engagement. Another main type of interaction involved learning from other sectors’ expertise, which could also avoid a duplication of efforts to build up expertise within each sector:

“So we don’t come into it trying to be health specialists, if you know what I mean. We’re coming in with a wildlife [perspective] and seeing how we can add that understanding to people and organisations that have an animal health or public health focus.”

(Key informant, environment)

Participants acknowledged that there were substantial barriers to inter-sectoral interaction, however. Typically, sectors had different goals, terminology, and metrics for success, as well as different governance structures. Some key informants saw the conflicting goals as a strength and an opportunity for learning:

“So it’s definitely a strength to work with people that do not have the same perspective and the same interest. The key thing is to define this common interest.”

(Key informant, other)

While all key informants acknowledged the importance of cross-sectoral interaction around the governance of wildlife trade and the prevention of emerging zoonoses, some acknowledged that working within a sector could sometimes be a more expedient way of making progress.

## Discussion

### Statement of principal findings

The network of transnational organisations focusing on the governance of wildlife trade is composed of many types of organisations from sectors including human, animal, and environmental health. Our findings highlight the intensely inter-sectoral nature of this area, and a desire among its members to see greater interaction. This interaction was supported by the establishment of network organisations and the efforts of a core, multi-sectoral group of organisations that was well-connected and influential within the transnational network as well as with national-level actors.

However, inter-sectoral interaction was often challenging due to conflicting aims and perspectives. The network was also impacted by power dynamics: the human health sector, while historically less involved in wildlife trade governance, was seen as better resourced, more powerful, and influential. This was seen as both a boon for the network and its interests, as the involvement of the human health sector brought resources and a larger platform, and a risk to equity between actors. A One Health approach was seen as a potential way to build decision-making and governance processes on a more equitable footing, but key informants emphasised the importance of clarity around goals and implementation for such an approach.

### Strengths and limitations

This network was conceptualised as a binary undirected graph based on participant responses about organisational interactions. This meant that connections identified by one organisation were taken to be mutual, and the intensity and more specific nature of relationships was not considered. This decision was made after piloting the survey instrument: we found that participants were unwilling to characterise the nature and frequency of professional interactions between their organisations’ many partners. The time taken to complete this more detailed survey instrument may have caused participants to give up before completing the survey, a comment explicitly made in some survey responses. We therefore simplified the survey instrument and analysis approach to look at interactions more broadly defined, characterised simply as existent or non-existent. As a result, our quantitative analysis could not incorporate more nuanced aspects of organisational relationships. Our characterisation of ‘interactions’ therefore conceals complexity: interactions may have consisted of activities including resource-sharing, information sharing, or providing and securing agreement, and even included interactions that may have been antagonistic, as well as collaborative. However, by complementing the survey data with qualitative data, we were able to explore aspects of network process and function that could not be captured through the survey, which helped to capture nuance which might have been missed if focusing only on the quantitative analysis. For example, our quantitative results suggested that organisations were not more likely to be connected to organisations within their own sector compared to other sectors. However, our qualitative analysis highlighted that while cross-sectoral connections existed, the nature and intensity of these connections did vary by sector.

Our study also faced two of the key challenges which are typical of organisational network analysis. First, while the analysis was carried out at the level of organisations, our participants were individuals working within those organisations, targeting a key informant in each organisation in line with typical practice in organisational network analysis [41–43]. It is therefore possible that their perspectives did not include all of their organisations’ interactions [44]. We tried to mitigate this by emphasising that we were interested in the interactions of the organisation as a whole; by inviting potential participants to forward the survey to a colleague who might be best placed to speak from the organisational perspective, if they did not feel able to do so; and by advising participants that they could gather input from colleagues to complete the survey if they felt this was appropriate.

Second, given that we received responses from less than half of the organisations identified in the network, our data set is likely to be characterised by data missingness, which is a key challenge in survey-based research, and network analysis in particular [29, 44]. At the data collection phase, we attempted to mitigate this by following up with participants up to three times over the space of several months; seeking personalised introductions; and inviting additional participants from the same organisations where no response was received. At the analysis phase, we included inter-organisational ties where they were confirmed by a single organisation, in order to minimise the impact of missing data on our findings. The centrality measures discussed here, and other node-level characteristics, are sensitive to missing data, and higher amounts of missing data lead to lower correlation between the ‘real’ and measured properties of nodes, though this effect may vary across networks with different properties [45]. In this analysis, measured centrality of groups of organisations with lower response rates, namely organisations in the human and environmental health sectors and non-governmental organisations, may therefore be less accurate than measured centrality for other groups. However, it is not known whether this would have led to an over- or under-estimation of organisation centrality: one of the under-represented groups, the environmental sector, was found to be highly central, while the remaining groups were not. Our mixed methods approach also adds depth to our understanding, allowing us to interrogate the findings from the quantitative analysis, identifying points where both data sets are aligned and divergent.

Finally, network analysis provides a snapshot of a network at a given point in time. While the mixed methods approach allowed us to understand how the network had been evolving, particularly during the COVID-19 pandemic, the network has most likely continued to evolve since data collection ended.

### Implications for policy and practice

In this analysis, we found that the COVID-19 pandemic, and other zoonotic epidemics and pandemics, played a key role in fostering greater multi-sectoral interaction across this network. As our qualitative findings suggest that, while organisational interactions happened across sectors, inter-sectoral work is challenging, that working within one sector may be more expedient, and that bureaucratic barriers to agile action exist at this level of governance, the importance of building cross-sectoral governance structures in ‘peace time’ – when international health crises are not driving urgent action – seems clear.

The importance of inter-sectoral work and the value of a One Health approach to the governance of wildlife trade and the prevention of emerging zoonoses seems broadly recognised by our key informants. However, participants stressed that this must be more than a rhetorical position, and must be supported by clear goals and strategies for implementation. Our findings highlight that, while the organisations in this network are broadly in agreement with the importance of One Health, they may have strongly conflicting views around what the term means and what the approach looks like in practice.

While this analysis focused on the transnational level of governance, the interplay between these actors and national and subnational governments is key to making recommendations based on these findings. With the exception of the treaty secretariats that featured in the network, most of the organisations do not have a power to compel, and instead act by convening different parties, developing standards and providing information and expertise. Within these boundaries, the organisations within this network can build on their successful practice of sharing information, identifying and connecting experts, developing guidance, and convening countries for mutual learning. However, it would be important to build the inter-sectoral, One Health approach to these issues into their interactions with national governments, as this could also diffuse the approach to the national and sub-national level.

### Comparison to existing literature

To our knowledge, this is the first global network analysis focused on the governance of wildlife trade within the context of prevention of emerging zoonoses by transnational organisations, though a country-level network analysis of UK-based organisations combatting the illegal wildlife trade was published pre-pandemic [46]. While some international actors were identified as belonging to this country-level network, these were much more focused on conservation, animal welfare and crime prevention (e.g., Interpol, International Institute for Environment and Development, International Fund for Animal Welfare, World Wildlife Fund). Organisations focused on agriculture and food security, which were identified as part of the network in the current study, were not identified. This may reflect the UK-focused study’s emphasis specifically on *illegal* wildlife trade, which may not be considered for its contribution to food security in the same way, as well as its lack of emphasis on zoonotic disease, which centres interactions between wildlife, livestock, and people – including in food-relevant sites such as farms and markets – as potential drivers of disease transmission.

Health-focused network analyses of global actors are also relatively rare, although an existing analysis of the global health space more broadly found similar types of actors involved, including non-governmental organisations, inter-governmental organisations, professional associations and national governments with an international remit [23]. In contrast to this analysis, we did not identify any industry bodies as being a part of this network. While key informants mentioned the relevance of certain industry sectors, such as agriculture and pharmaceutical development, to this area, these actors were not named as being directly part of the network. The wildlife trade itself did not seem to have a representative industry body with ties to this network. At other levels of governance, our findings were aligned with other network analysis studies which have highlighted the wide range of sectors involved in the control of zoonotic disease, and highlight the role of disease outbreaks in fostering greater inter-sectoral interaction and more openness to a One Health approach [47].

Our findings align with previous studies on barriers to effective One Health efforts. Existing literature also highlights some of the issues identified in our study, including professional divisions between human, animal and environmental health practitioners and policymakers; differences in terminology; and a lack of coordination and collaboration [48–50]. However, in contrast to studies focused on One Health topics more broadly, which often find the environmental health sector to be under-represented in One Health efforts [49, 51], this sector was fairly central to our network, perhaps because of the network’s focus on wildlife, and particularly the wildlife trade, as opposed to domesticated animals. While several recent disease outbreaks have been linked to changing human-wildlife interactions [10, 11], wildlife and wildlife trade predominantly fall under the remit of environmental protection, hence environmental health, with much global governance of wildlife being focused on conservation [12, 52]. Indeed, the wildlife trade, both legal and illegal, has been identified as one of the major drivers of biodiversity loss, which has corresponded with policy responses at various governance levels [52–54]. Our analytical approach also yielded new insights around the relative centrality of the human health sector in this particular One Health topic: while many of the organisations in the network belonged to the human health sector, the position of these organisations within the network seemed to suggest that it may be less central to the network.

Finally, our study seems to confirm findings from similar studies on network governance in global health, which have emphasised the importance of lead organisations within networks to generate policy momentum. When studying organisational networks, an essential question is how the collaboration between actors in these systems is governed. Provan and Kenis proposed three ways in which organisational networks could be governed: through ‘shared governance’, with wide-spread distribution of power and decision-making functions; through ‘lead organisation governance’, with one member organisation leading governance; or through ‘network administrative organisation governance’, with a dedicated network administrative organisation to govern the network [55]. Our network does not seem to fit neatly into any of the three categories, but most closely resembles a shared governance approach in which the network is governed by all organisations interacting with each other, resulting in a somewhat decentralised network, though a core group of organisations was central to network governance. Inferring from studies of network governance focused on other global health topics [56], a dedicated network administrative organisation for wildlife trade might be an important step towards improving network governance in this policy space, which may also improve its effectiveness.

### Future research

While our quantitative analysis was cross-sectional, qualitative findings highlighted the impact the COVID-19 pandemic was continuing to have on this network. Additional research could continue to explore how this network is changing over time; for example, by taking a longitudinal network analysis approach [44]. This approach is useful when evaluating the effectiveness of interventions to strengthen interaction and connection across the network, and could be used to evaluate the impact of mechanisms to strengthen interaction, such as the ‘network organisations’ that we identified.

In addition, research is needed to better understand how cross-sectoral interactions, such as those occurring in this network, can strengthen relational coordination, defined as “a mutually reinforcing process of communicating and relating for the purpose of task integration” [57]. A survey tool can be used to evaluate the extent of frequent, timely, accurate, and problem-solving communications and to gauge the level of shared goals, shared knowledge, and mutual respect required for effective collaborative initiatives [58]. Structures that support greater relational coordination can help network actors to think about working towards shared goals and understanding how their own contributions and those of others contribute to shared outcomes [57]. Stronger relational coordination may therefore contribute to more effective action in complex networks such as the one analysed in this study, where competing goals can impede progress. Assessing and improving relational coordination can help reframe the discussion around risk mitigation and the need to revise existing policy, considering the expertise, competencies, and experiences of cross-sectoral networks.

Finally, further research could assess to what extent the current level of interaction in this network supports or undermines these organisations’ capacity to prevent the emergence of zoonoses in human and animal populations. While key informants seemed to broadly share the assumption, common in network-focused literature [59], that more interaction across this network would support more effective action, this assumption could be further explored empirically. Multi-sectoral collaboration is often seen as positive, especially for complex problems where bringing together knowledge and resources can support new ways of thinking and the implementation of more effective solutions, particularly where action from multiple actors may be needed to solve a problem [59]. However, these interactions come at a cost in terms of time and resources [60], and the extent to which potential benefits outweigh costs should be assessed.

## Conclusion

The network of transnational organisations focused on the governance of wildlife trade is highly multi-sectoral, but barriers still exist to inter-sectoral interaction.

This study highlights some important ways in which the network can be strengthened, including by continuing to build and invest efforts into multi-sectoral governance and action in a sustained way, moving from a more reactive stance to one focused on prevention and preparedness. This will require the continued support of the human health sector, which has sometimes seen issues, such as wildlife health, as beyond its remit. Transnational organisations may also explore options for connecting with country-level partners in a more cross-sectoral way, helping to mainstream a One Health approach to policy and governance. Finally, a One Health approach to governance at this level, which has gained traction throughout the COVID-19 pandemic, may be a mechanism to support more equitable balancing of roles and agendas in this space. However, this must involve agreement around priorities and clear goal setting to support effective action.

### Electronic supplementary material

Below is the link to the electronic supplementary material.

Supplementary Material 1

## Data Availability

Because the global governance community interviewed as part of this study is relatively small and closely knit, full interview transcripts cannot be shared beyond the project team as this may compromise participants’ anonymity. The anonymised quantitative data set is available from the corresponding author on reasonable request. Analysis code for the quantitative analysis is available on GitHub.

## Abbreviations

MMSNA: Mixed methods social network analysis
