# Supplementary material for: Governance of the wildlife trade and the prevention of emerging zoonoses: a mixed methods network analysis of transnational organisations, silos, and power dynamics
Source: Global Health. 2024 Jun 20;20:49. doi: 10.1186/s12992-024-01055-7 (PMC11188226; doi:10.1186/s12992-024-01055-7)
Supplement: Supplementary file 1 — Supplementary Material 1 [file 12992_2024_1055_MOESM1_ESM.docx]

**Supplementary file 1**

Table S1: Centrality measures for all organisations in the network

| Organisation ID | Sector | Type | Degree | Closeness | Betweenness |
| --- | --- | --- | --- | --- | --- |
| 1 | Animal health | Inter-governmental organisation | 30 | 0.00833333 | 240.878462 |
| 2 | Human Health | Inter-governmental organisation | 16 | 0.00769231 | 69.4178663 |
| 3 | Other | Inter-governmental organisation | 3 | 0.00628931 | 0 |
| 4 | Other | Inter-governmental organisation | 23 | 0.0078125 | 159.317501 |
| 5 | Environmental health | Treaty secretariat | 19 | 0.0078125 | 193.617524 |
| 6 | One Health | Research institution | 20 | 0.00840336 | 215.273201 |
| 7 | Environmental health | Inter-governmental organisation | 19 | 0.00763359 | 62.3341022 |
| 8 | Environmental health | Inter-governmental organisation | 28 | 0.00833333 | 560.930942 |
| 9 | Environmental health | Treaty secretariat | 12 | 0.00684932 | 7.63469863 |
| 10 | Other | Inter-governmental organisation | 20 | 0.00833333 | 216.162039 |
| 11 | Other | Inter-governmental organisation | 8 | 0.00606061 | 1.16829004 |
| 12 | Other | Inter-governmental organisation | 15 | 0.00704225 | 24.7723124 |
| 13 | Other | Inter-governmental organisation | 3 | 0.00564972 | 0 |
| 14 | Other | Trade association | 5 | 0.0060241 | 1.71798419 |
| 15 | Environmental health | Non-governmental organisation | 14 | 0.00746269 | 69.0562221 |
| 16 | Animal health | Non-governmental organisation | 4 | 0.00588235 | 0.11111111 |
| 17 | Other | Inter-governmental organisation | 1 | 0.00534759 | 0 |
| 18 | Other | Research institution | 11 | 0.00684932 | 25.5696321 |
| 19 | Animal health | Network | 21 | 0.00775194 | 70.1597161 |
| 20 | Other | Research institution | 3 | 0.00613497 | 0 |
| 21 | Environmental health | Treaty secretariat | 16 | 0.00735294 | 27.6264184 |
| 22 | Environmental health | Non-governmental organisation | 21 | 0.00740741 | 110.174628 |
| 23 | Animal health | Non-governmental organisation | 12 | 0.00735294 | 76.03878 |
| 24 | Animal health | Non-governmental organisation | 5 | 0.00609756 | 0.42539683 |
| 25 | Animal health | Professional association | 5 | 0.00636943 | 25.2323232 |
| 26 | Animal health | Research institution | 10 | 0.00645161 | 67.9253968 |
| 27 | Environmental health | Non-governmental organisation | 22 | 0.0078125 | 143.458116 |
| 28 | Human Health | Government department | 6 | 0.00628931 | 2.61377789 |
| 29 | One Health | Network | 19 | 0.00746269 | 411.478703 |
| 30 | Environmental health | Non-governmental organisation | 8 | 0.00636943 | 0.91313131 |
| 31 | One Health | Network | 7 | 0.00621118 | 1.08968254 |
| 32 | One Health | Network | 8 | 0.00598802 | 2.63571429 |
| 33 | Animal health | Professional association | 15 | 0.00675676 | 394.320635 |
| 34 | One Health | Non-governmental organisation | 4 | 0.00591716 | 0 |
| 35 | Animal health | Research institution | 4 | 0.00598802 | 0.09090909 |
| 36 | Other | Non-governmental organisation | 9 | 0.00617284 | 325.431818 |
| 37 | Environmental health | Non-governmental organisation | 9 | 0.00662252 | 1.8465368 |
| 38 | Other | Professional association | 5 | 0.00598802 | 2.57642857 |
| 39 | Other | Research institution | 1 | 0.00512821 | 0 |
| 40 | Other | Regional economic initiative | 1 | 0.00512821 | 0 |
| 41 | Other | Research institution | 1 | 0.00534759 | 0 |
| 42 | One Health | Network | 1 | 0.00534759 | 0 |
| 43 | One Health | Voluntary partnership secretariat | 5 | 0.00558659 | 198 |
| 44 | Human Health | Research institution | 1 | 0.00534759 | 0 |
| 45 | Environmental health | Non-governmental organisation | 2 | 0.00568182 | 0 |
| 46 | Human Health | Professional association | 1 | 0.00406504 | 0 |
| 47 | Environmental health | Non-governmental organisation | 1 | 0.00406504 | 0 |
| 48 | Human Health | Professional association | 1 | 0.00406504 | 0 |
| 49 | Environmental health | Voluntary partnership secretariat | 1 | 0.0049505 | 0 |
| 50 | Environmental health | Research institution | 1 | 0.0045045 | 0 |
| 51 | Other | Research institution | 1 | 0.00512821 | 0 |
| 52 | One Health | Network | 3 | 0.00571429 | 0 |
| 53 | Environmental health | Non-governmental organisation | 1 | 0.00497512 | 0 |
| 54 | Environmental health | Network | 1 | 0.00497512 | 0 |
| 55 | Human Health | Non-governmental organisation | 1 | 0.00497512 | 0 |
| 56 | Human Health | Consultancy | 1 | 0.00497512 | 0 |
| 57 | Environmental health | Non-governmental organisation | 1 | 0.00497512 | 0 |
| 58 | Environmental health | Research institution | 1 | 0.00497512 | 0 |
| 59 | Human Health | Professional association | 1 | 0.00465116 | 0 |
| 60 | Animal health | Non-governmental organisation | 1 | 0.00465116 | 0 |
| 61 | Human Health | Inter-governmental organisation | 1 | 0.00465116 | 0 |
| 62 | Human Health | Non-governmental organisation | 1 | 0.00465116 | 0 |
| 63 | Animal health | Non-governmental organisation | 1 | 0.00465116 | 0 |
| 64 | Animal health | Professional association | 1 | 0.00465116 | 0 |
| 65 | Environmental health | Non-governmental organisation | 1 | 0.00436681 | 0 |
| 66 | Environmental health | Non-governmental organisation | 1 | 0.00436681 | 0 |
| 67 | Environmental health | Non-governmental organisation | 1 | 0.00436681 | 0 |
| 68 | Environmental health | Non-governmental organisation | 1 | 0.00436681 | 0 |
| 69 | Environmental health | Non-governmental organisation | 1 | 0.00436681 | 0 |
